# Deciphering the Morphological Correlation Between Stature and External Ear: An investigative approach in the field of Forensic Science

**DOI:** 10.12688/f1000research.164138.1

**Published:** 2025-07-07

**Authors:** M Shama das, Vinod C Nayak, Pratima R Bhat, Lahari U, Alana Chacko

**Affiliations:** 1 Department of Forensic Medicine and Toxicology, Kasturba Medical College, Manipal Academy of Higher Education, Manipal, Karnataka, 576104, India; 2 Department of Community Medicine, Kasturba Medical College, Manipal Academy of Higher Education, Manipal, Karnataka, 576104, India

**Keywords:** Forensic Anthropology, Personal identification, Ear morphology, Stature, Linear regression, Correlation, Estimation of stature.

## Abstract

Abstract: Forensic anthropology is the study of skeletal remains for the identification of individuals and anthropology deals with study of human. Many research has been conducted to estimate stature from different body parts like foot, extremities, hand, vertebrae etc. In some cases, finding all body parts and bones in whole will be difficult in the crime scene for forensic examination. In such cases it becomes necessary to use other region such as the head and facial region for the estimation of height. Determination of height is an important in forensic examination for identification of the individual. This study was done to find the correlation between the stature and the morphological variations of the external auricular region for the estimation of stature for the purpose of forensic investigation. The study was conducted on 385 male individuals, total of 4 ear parameters were measured from both the ears using the digital caliper. The data was analyzed using Jamovi version 2.4.11. The result showed that there was positive correlation between all the dimensions with stature. The linear regression was done to find the statically significant difference between the stature and the parameters and it was found that the most predicted variable in males is the left ear length(p<0.001). This study concludes that the external auricular morphometry can be as an additional tool for the estimation of stature in the forensic investigation.

## Introduction

Personal identification basically refers to the process of determining an individual’s unique identity. A crucial component of forensic science is human identification. Knowing the victim’s identification is the first step in gathering information about them. In the living, identification relies upon the district morphological features which are distinct to each person. In the case of bones, the process becomes complicated and it demands more meticulous examination of the remains. ^1^

The measurement of living body proportions in order to understand physical variances is known as anthropometry. ^2^^,^^3^ The main aspects of identification of an individual are age, sex, and stature. ^4^^–^^6^ The assessment of stature from various bodily parts, including the hands, trunk, foot, extremities, and vertebrae, has been the subject of numerous research. ^7^ Given the possibility that not all of these body parts would be found at the crime scene for forensic investigation, it becomes essential to utilize other body parts like the cranial and facial region for identification purpose. ^1^^,^^8^^,^^9^

Human ears are the most distinctive facial characteristics, and their structure can reveal an individual’s age and sex. ^10^^–^^12^ An effective method for estimating stature is the examination of ear morphological dimensions like auricle dimension and pinna breadth and measurements of ear lobular span and ear lobule width. ^13^^–^^15^

**Figure 1.**
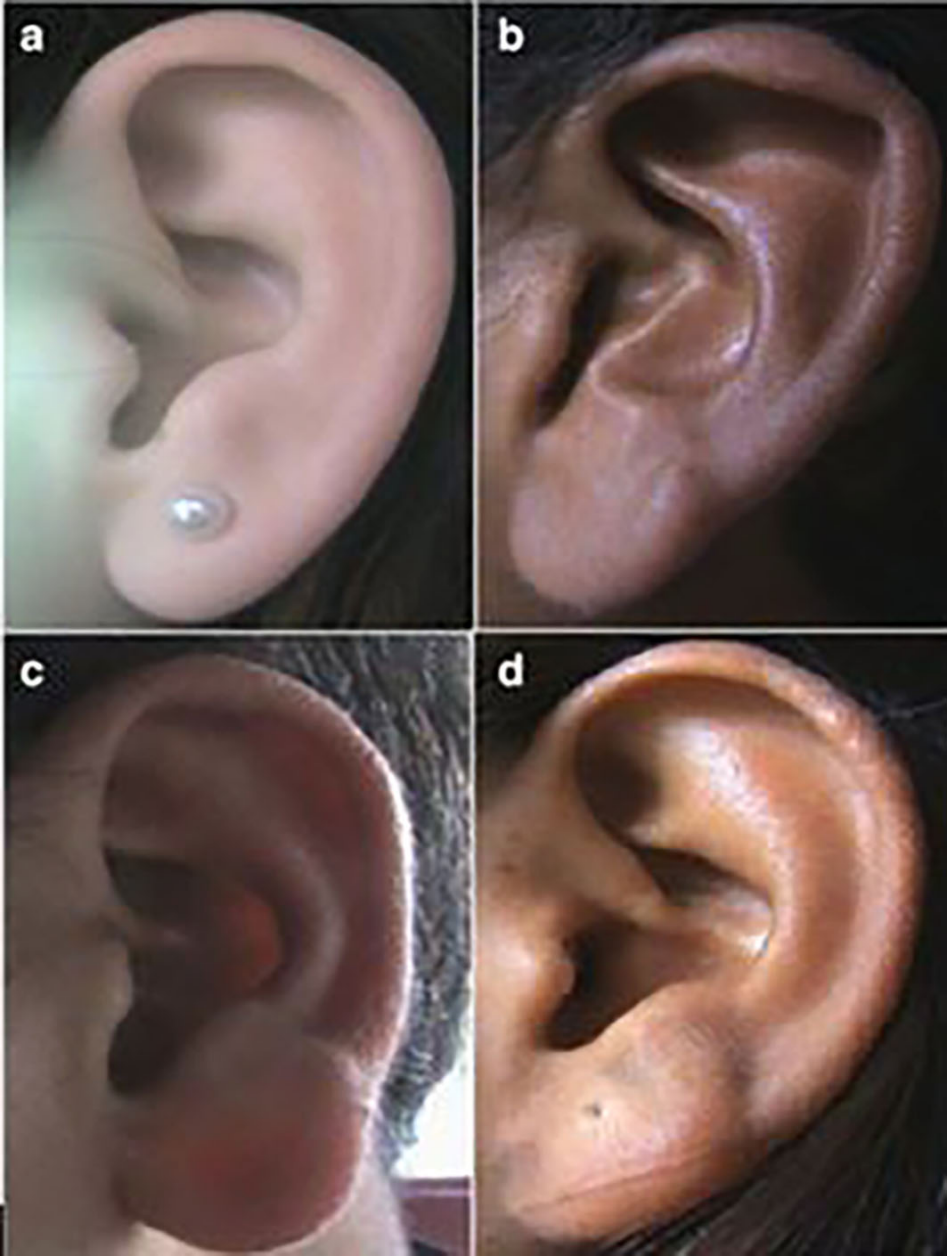
Different types of ear shapes: (a) Oval, (b) Triangular, (c) Rectangular, (d) Round.

**Figure 2.**
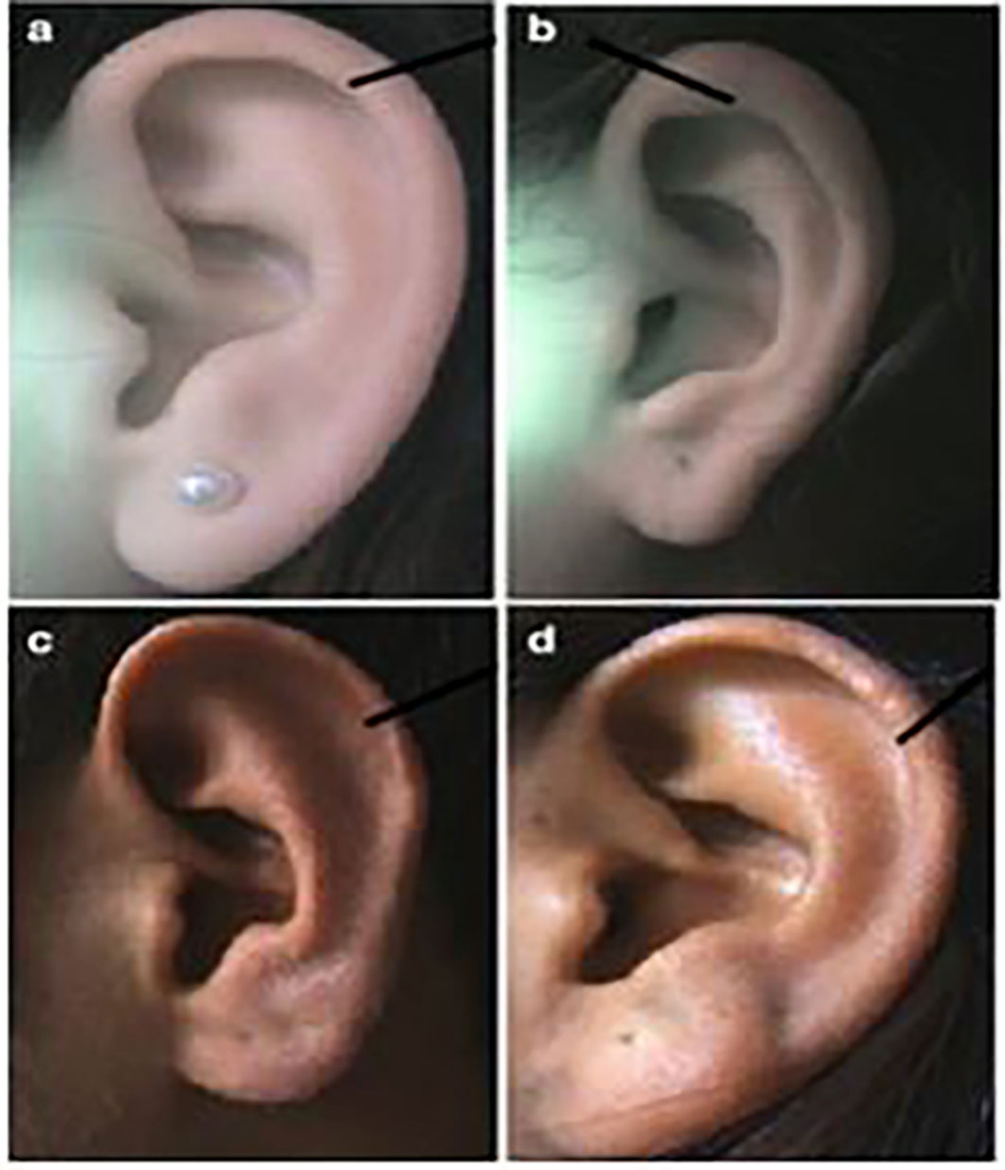
Different forms of helix: (a) normally rolled, (b) wide covering scapha, (c) flat, (d) concave marginal.

**Figure 3.**
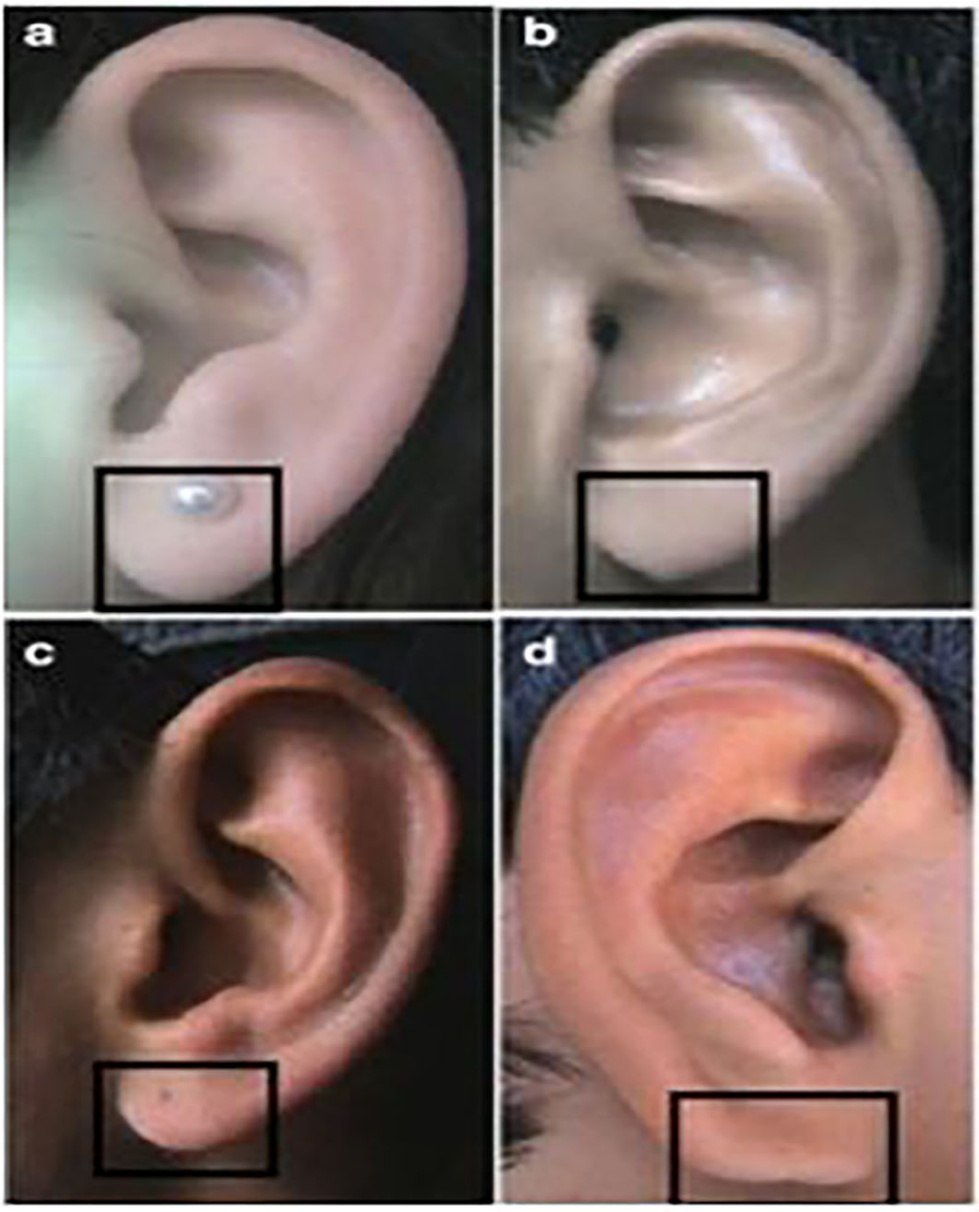
Different types of lobule shapes: (a) Tongue, (b) Triangular, (c) Arched, (d) Square.

**Figure 4.**
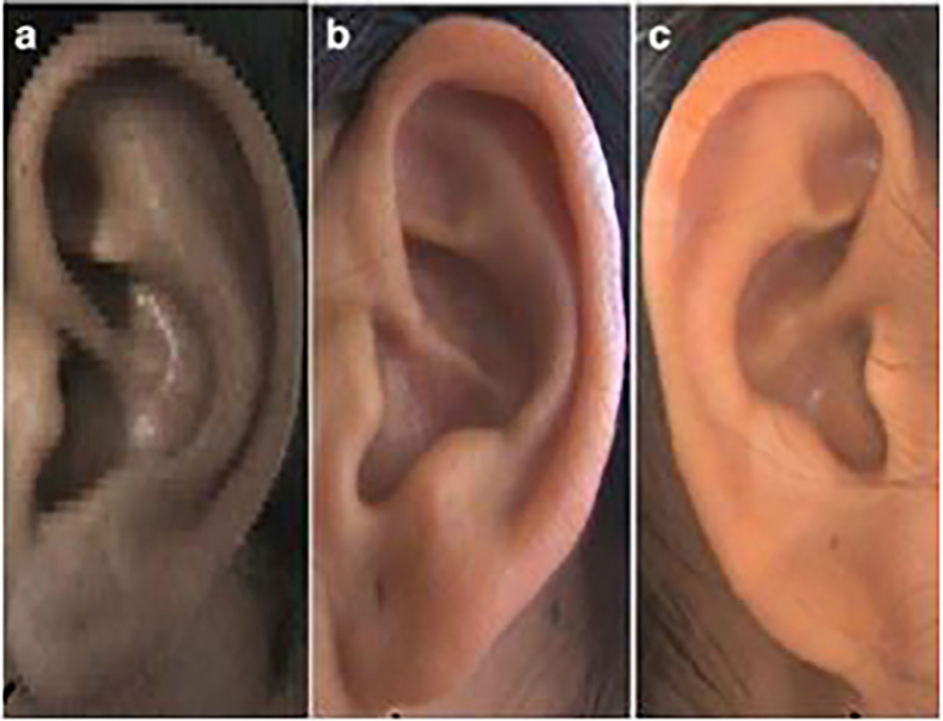
Different types of ear lobe attachment: (a) Free, (b) Partially attached, (c) Attached.

Some research papers have shown varied ear shapes, ^16^ varied types of helixes and tragus, classification of Darwin’s tubercles, and varied types of ear lobes. The types of ear shape are oval, triangular, rectangular, round. The helix of the ear comes in various shapes, including flat, gently curved at the edges, broadly covering the scapha, or the more common rolled form. The different lobule shapes are tongue, triangular, arched, square. The various types of earlobe attachment are classified as free, partially attached, attached ^17^^–^^20^ ( Figures 1, 2, 3, 4).

Many research papers have found that external ear proportions are uniformly varied in individuals, the external ear dimensions are considerably larger in males in addition, ^21^ the ear shows a bilateral asymmetry. Few studies also show that the configuration and proportion of ear can show craniofacial reconstruction in forensic examination. ^22^ There is a association of stature and external ear measurements, according to numerous research.

So, the goal of the study is to find the correlation between the external ear measurements and height and also to find the morphological variation between each individual.

## Methods

The study was done on 385 adult male individuals, ^29^ between the age 18-50 years. The participants were students and professionals of MAHE, Manipal. Total of 8 measurements were taken from both ears, the measurements were Auricle length, Auricle breadth, Lobule length and Lobule breadth using standard caliper and stature was taken using measuring tape. Participants with deformed ear and female participants (wearing earrings) and also male participants wearing earring are excluded. The written informed consent was taken from the participants before taking the measurements.

**Figure 5.**
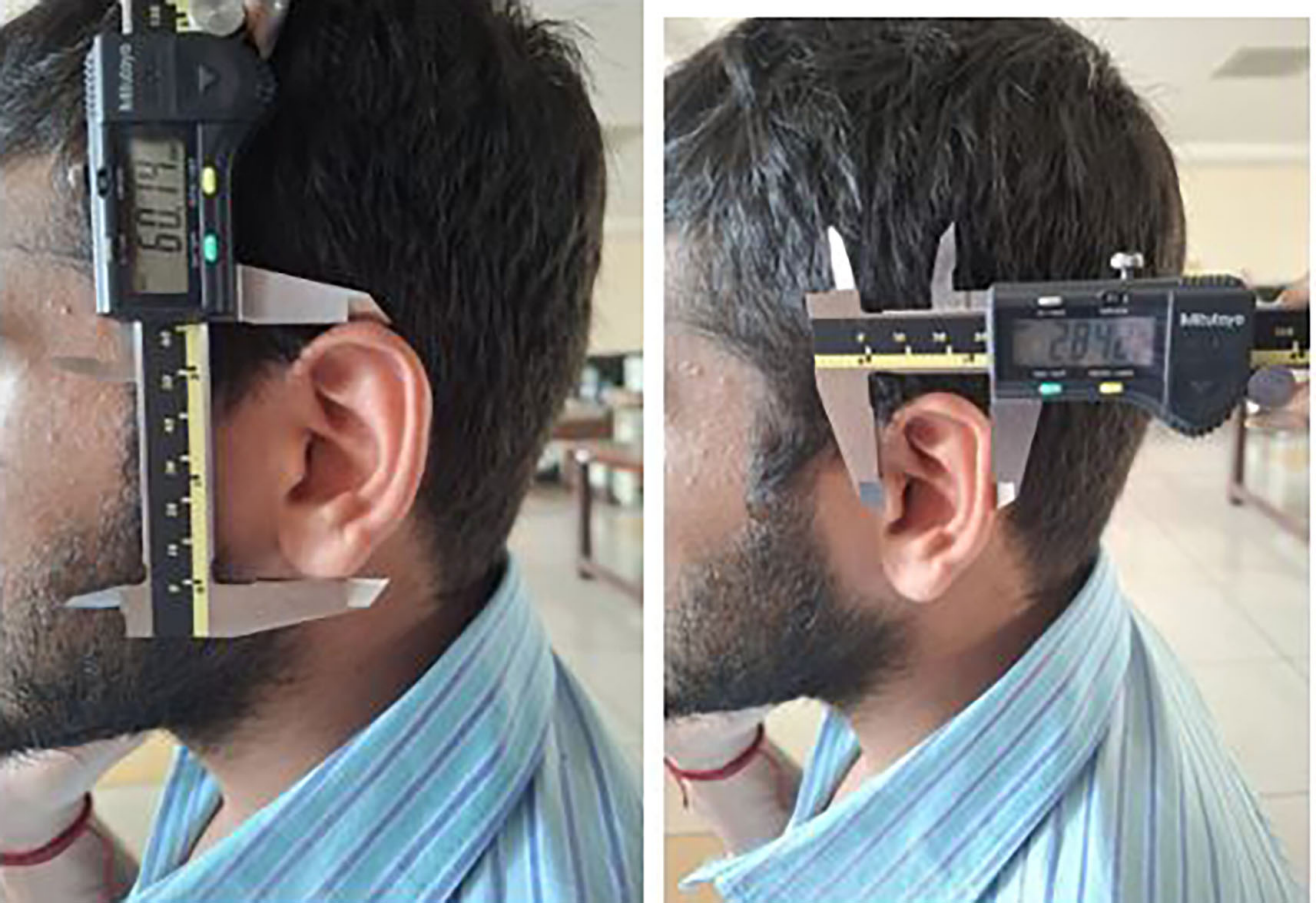
Measuring the ear length and ear width using digital caliper.

**Figure 6.**
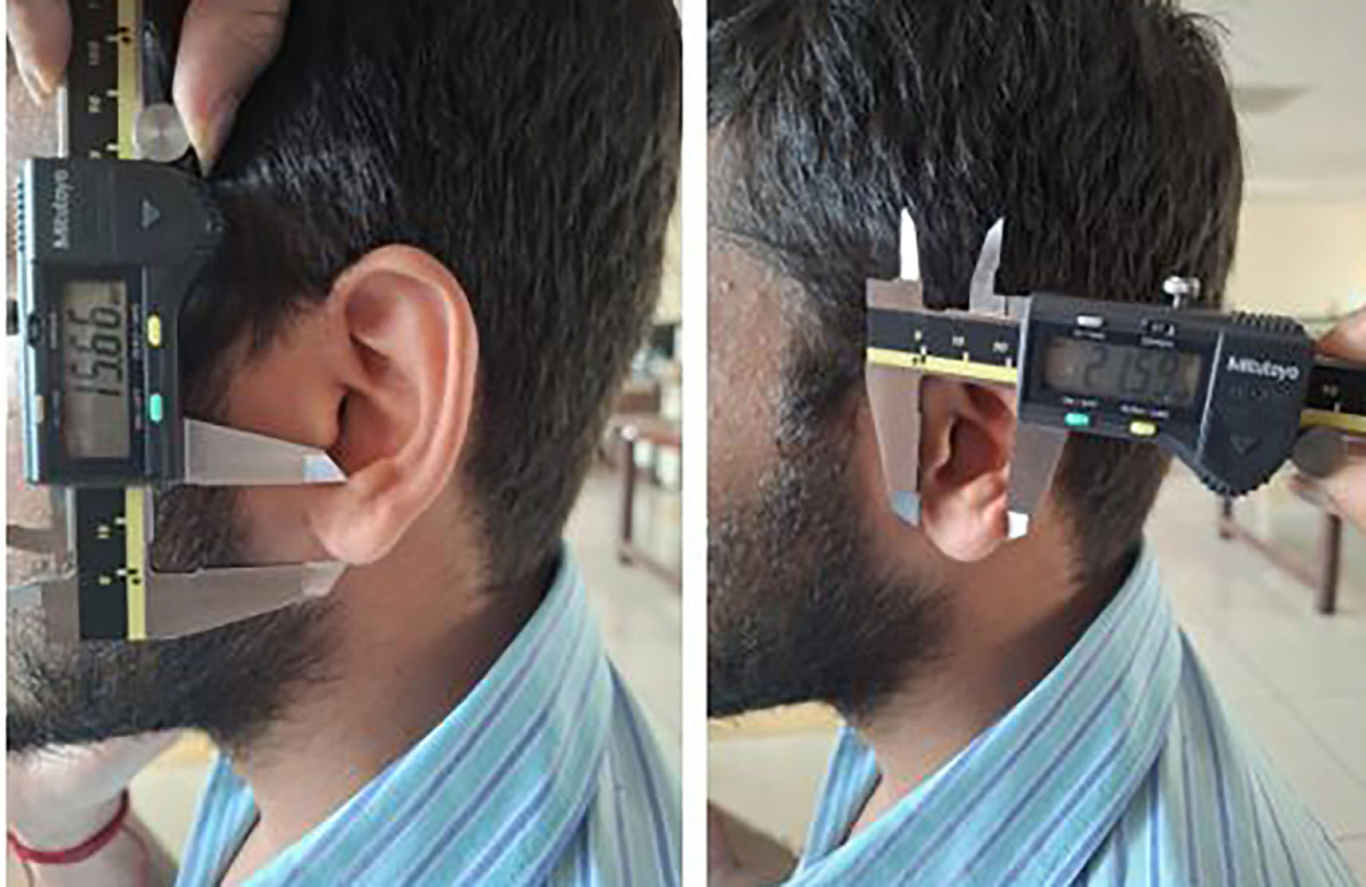
Measuring the lobule length and lobule width using digital caliper.

Each participant was made to stand barefoot, and the stature was measured using the measuring tape. The participants were made to sit and then the 8 measurements were taken using the digital calliper ( Figures 5, 6).

The data was analysed using Jamovi version 2.4.11. The mean and standard deviation were calculated for all the parameters. Correlation between the stature were tested using spearman’s rank correlation. The linear regression was used to find the statistically significant difference between all the parameters and stature.

## Result

From Table 1, the mean and standard deviation of different parameters are age (24.7 ± 6.78), stature (172 ± 6.90), left ear length (6.14 ± 0.462), left ear width (3.22 ± 0.321), left lobule length (2.16 ± 0.350), left lobule width (1.99 ± 0.334), right ear length (7.55 ± 28.6), right ear width (3.24 ± 0.305), right lobule length (2.07 ± 0.336), right lobule width (1.98 ± 0.330). Here the right ear parameters show higher mean and standard deviation then the left ear parameters, except for the left lobule length and left lobule width which is higher than right lobule length and width.

**Table 1.** Descriptive statistics of different parameters (n=385).

PARAMETERS	Mean ± SD
AGE	24.7 ± 6.78
STATURE	172 ± 6.90
LEFT EAR LENGTH	6.14 ± 0.462
LEFT EAR WIDTH	3.22 ± 0.321
LEFT LOBULE LENGTH	2.16 ± 0.350
LEFT LOBULE WIDTH	1.99 ± 0.334
RIGHT EAR LENGTH	7.55 ± 28.6
RIGHT EAR WIDTH	3.24 ± 0.305
RIGHT LOBULE LENGTH	2.07 ± 0.336
RIGHT LOBULE WIDTH	1.98 ± 0.330

SD = Standard Deviation n = no of individuals.

From the Table 2, the result show that in males, the left ear length (R ^2^= 0.0780, p<0.001), left lobule length (R ^2^= 0.00734, p=0.093), left lobule width (R ^2^= 0.0112, p=0.038), right ear length (R ^2^= 0.0660, p=0.072), right ear width (R ^2^= 0.0144, p=0.019), right lobule length (R ^2^= 0.00886, p=0.065), right lobule width (R ^2^= 0.0157, p=0.014) and left ear width (R ^2^=0.00380, p=0.227), among all these parameters left ear length, right ear length shows a statistically significant difference between the stature.

**Table 2.** Statistical analysis of different parameters using linear regression (n=385).

PARAMETERS	R	R ^2^	t	p-value
LEFT EAR LENGTH	0.279	0.0780	5.69	<0.001 *
LEFT EAR WIDTH	0.0617	0.00380	1.21	0.227
LEFT LOBULE LENGTH	0.0857	0.00734	1.68	0.093
LEFT LOBULE WIDTH	0.106	0.0112	2.08	0.038
RIGHT EAR LENGTH	0.257	0.0660	1.84	0.072
RIGHT EAR WIDTH	0.120	0.0144	2.36	0.019
RIGHT LOBULE LENGTH	0.0941	0.00886	1.85	0.065
RIGHT LOBULE WIDTH	0.125	0.0157	2.47	0.014

* p value <0.05- Significant.

From the Table 3, the result show that in males, the left auricle length (r= 0.279), left ear width (r= 0.109), left lobule width (r= 0.113), right auricle length (r= 0.279), right ear width (r= 0.130), right lobule width (r= 0.120) shows moderate positive correlation with the stature, while the left lobule length (r= 0.096), right lobule length (r= 0.077) shows weak positive correlation with the stature.

**Table 3.** Statistical analysis of different parameters using Spearman’s rank correlation (data is not normally distributed) (n=385).

PARAMETERS	Spearman’s coefficient (r)	p-value
LEFT EAR LENGTH	0.279	<0.001 *
LEFT EAR WIDTH	0.109	0.033
LEFT LOBULE LENGTH	0.096	0.059
LEFT LOBULE WIDTH	0.113	0.027
RIGHT EAR LENGTH	0.279	<0.001 *
RIGHT EAR WIDTH	0.130	0.010
RIGHT LOBULE LENGTH	0.077	0.130
RIGHT LOBULE WIDTH	0.120	0.019

* p value <0.05- Significant.

From the Table 4, the mean age is 23 years which is taken from the 385 individuals. Then the mean age is divided into two groups that is lesser than or equal to 23 years of age and greater than or equal to 23 years of age. The two age groups are taken for the 8 parameters. The Median [IQR] value is taken for all the 8 parameters between the two-age group to find which parameter has significant difference among the individuals based on different age. Among the age ≤23, the left ear length (6.14[5.65-6.31]), left ear width (3.31[3.19-3.39]), left lobule length (1.97[1.81-2.24]), left lobule width (2.05[1.86-2.18]), right ear length (6.06[5.55-6.17]), right ear width (3.43[3.11-3.49]), right lobule length (1.95[1.81-2.20]), right lobule width (2.16[1.80-2.27]) and among the age ≥23, the left ear length (6.21[5.92-6.40]), left ear width (3.29[3.10-3.50]), left lobule length (2.19[1.90-2.35]), left lobule width (2.15[2.02-2.32]), right ear length (6.14[5.76-6.32]), right ear width (3.29[3.15-3.45]), right lobule length (2.04[1.87-2.43]), right lobule width (2.20[2.01-2.31]).

**Table 4.** Result comparing all parameters between each individual with two age group using Mann-Whitney U test (non-normally distributed).

PARAMETERS	AGE	Median[IQR]	p-value
LEFT EAR LENGTH	≤23	6.14[5.65-6.31]	0.371
	≥23	6.21[5.92-6.40]	
LEFT EAR WIDTH	≤23	3.31[3.19-3.39]	0.927
	≥23	3.29[3.10-3.50]	
LEFT LOBULE LENGTH	≤23	1.97[1.81-2.24]	0.115
	≥23	2.19[1.90-2.35]	
LEFT LOBULE WIDTH	≤23	2.05[1.86-2.18]	0.016
	≥23	2.15[2.02-2.32]	
RIGHT EAR LENGTH	≤23	6.06[5.55-6.17]	0.329
	≥23	6.14[5.76-6.32]	
RIGHT EAR WIDTH	≤23	3.43[3.11-3.49]	0.623
	≥23	3.29[3.15-3.45]	
RIGHT LOBULE LENGTH	≤23	1.95[1.81-2.20]	0.120
	≥23	2.04[1.87-2.43]	
RIGHT LOBULE WIDTH	≤23	2.16[1.80-2.27]	0.238
	≥23	2.20[2.01-2.31]	

IQR- Inter Quartile Range.

Based on the result, the right ear length and left lobule width shows significant difference among the individuals with respect to the age. The result shows that as age increases the right ear length and left lobule width will show significant difference from person to person.

## Discussion

Human identification is very important in many traumatic events. The main aim of forensic anthropologist when dealing with bones is to find out the gender, height, years of living and race. ^23^^,^^24^ The determination of height is necessary in forensic investigation in cases like mutilated human remains, highly decomposed body. There are several benefits of using somatological dimensions of outer ear for identification purpose. ^19^ Therefore, the goal of the research was to estimate the stature from the morphological variation of external auricle and also to evaluate the morphological differences among persons.

Abdelaleem SA et al., ^25^ their paper found that there was a affirmitive correlation between all measurements of both ears and stature in both sexes. The study is not in agreement of our study. Archana Kumar et al., ^13^ their study revealed no significant difference for both auricle dimensions in males, which is not a part of this study. In our study, there showed a significant difference in left ear length and right ear length with stature.

The key findings of the paper are as follows:

Using the spearman’s rank corelation, the present study revealed that there is an affirmative relation between left auricular length (r=0.279, p<0.001) and right ear span (r=0.279, p<0.001) with height.

Estimation of height from the outer auricle dimensions in this paper was completed by using the linear regression analysis. The present study revealed that left ear length (R ^2^=0.078), p-value (<0.001) was the best predictable variable to determine the stature and this concordance with Taura MG et al., ^26^ where their findings revealed that left ear width (R ^2^=0.086) is the best outcome and also the right auricle length (R ^2^=0.082) and left ear length (R ^2^=0.074).

Using Mann-Whitney U test, the right ear length and left lobule width showed significant difference between the individuals based on the age. The right ear length for age group ≤23 (6.06[5.55-6.17]), ≥23 (6.14[5.76-6.32]) and left lobule width for the age group ≤23 (2.05[1.86-2.18]), ≥23 (2.15[2.02-2.32]) showed greater difference in the Median [IQR] between the two age group, this shows that right ear length and left lobule width will show morphological variation as the age increases and not due to random causes.

This study proves the significance of external ear measurement as a unique tool used in forensic investigation for the estimation of stature from the morphological variation of ear, in cases of mutilated body found in crime scene, personal identification is important and when other body parts are not available then using the ear measurements we can estimate the stature. ^27^^,^^28^ it could also be beneficial in different fields including forensic anthropology, forensic science.

### Limitations

In the study, only four parameters were taken from both ears. Manual error was present during the collection of samples. Further studies can be performed using more parameters like facial features can be included with the ear parameters. Larger population can be considered for future studies.

## Ethical considerations

The ethical approval IEC No. 397/2024 was granted by “The Institutional Ethics Committee (ICE) of Kasturba Medical College and Kasturba Hospital Manipal” on 13 ^th^ August 2024. The data were kept completely undisclosed and no participant identifiers are used. The CTRI approval number is CTRI/2024/10/075400.

## Consent statement

The written informed consent was taken from all the participants **.** No minores were included in the study.

## Data Availability

Figshare: Stature and external ear morphology. figshare. https://doi.org/10.6084/m9.figshare.28782302.v1. ^29^ Data are available under the terms of the Creative Commons Attribution 4.0 International license (CC-BY 4.0). Extended data: the project contains the following extended data
• Informed consent sheet • Participation information sheet Data can be accessed: Figshare: Stature and external ear morphology_PI and IC forms, https://doi.org/10.6084/m9.figshare.28855265.v1. ^30^ Data are available under the terms of the Creative Commons Zero “No rights reserved” data waiver (CC0 Public domain dedication).
