# Deciphering the Morphological Correlation Between Stature and External Ear: An investigative approach in the field of Forensic Science

**DOI:** 10.12688/f1000research.164138.3

**Published:** 2025-10-13

**Authors:** M Shama das, Vinod C Nayak, Pratima R Bhat, Lahari U, Alana Chacko

**Affiliations:** 1 Department of Forensic Medicine and Toxicology, Kasturba Medical College, Manipal Academy of Higher Education, Manipal, Karnataka, 576104, India; 2 Department of Community Medicine, Kasturba Medical College, Manipal Academy of Higher Education, Manipal, Karnataka, 576104, India

**Keywords:** Forensic Anthropology, Personal identification, Ear morphology, Stature, Linear regression, Correlation, Estimation of stature.

## Abstract

Forensic anthropology is the study of skeletal remains for the identification of individuals, and anthropology more broadly deals with the study of humans. Many studies have been conducted to estimate stature from different body parts, such as the foot, extremities, hand, and vertebrae. However, in some cases, recovering all body parts and bones in their entirety can be difficult at the crime scene for forensic examination. In such cases, it becomes necessary to use another region, such as the head and facial region, for the estimation of height. Determination of height is a crucial aspect of forensic examination for the identification of the individual. This study was done to find the correlation between the stature and the morphological variations of the external auricular region for the estimation of stature for the purpose of forensic investigation. The study was conducted on 385 male individuals a total of four ear parameters were measured from both ears using the digital caliper. The data was analysed using Jamovi version 2.4.11. The result showed that there was a positive correlation between all the dimensions with stature. The linear regression analysis revealed a statistically significant relationship, with the most predicted variable in males being the left ear length (p<0.001). This study concludes that the external auricular morphometry can be an additional tool for the estimation of stature in forensic investigation.

## Introduction

Personal identification basically refers to the process of determining an individual’s unique identity. A crucial component of forensic science is human identification. Establishing the victim’s identity is the first step in gathering information about them. In the living individuals, identification relies upon the distinct morphological features that are unique to each person. In the case of skeletal remains, the process becomes complicated and requires more meticulous examination. ^1^

The measurement of living body proportions in order to understand physical variances is known as anthropometry. ^2^^,^^3^ The main aspects of individual’s identification are age, sex, and stature. ^4^^–^^6^ The assessment of stature from various bodily parts, including the hands, trunk, foot, extremities, and vertebrae, has been the subject of numerous studies. ^7^ Given the possibility that not all of these body parts may be found at the crime scene for forensic investigation, it becomes essential to utilize other body parts like the cranial and facial region for identification purposes. ^1^^,^^8^^,^^9^

Human ears are the most distinctive facial characteristics, and their structure can reveal an individual’s age and sex. ^10^^–^^12^ An effective method for estimating stature is the examination of ear morphological dimensions, such as auricle dimension and pinna breadth, as well as measurements of ear lobular span and ear lobule width. ^13^^–^^15^

**Figure 1.**
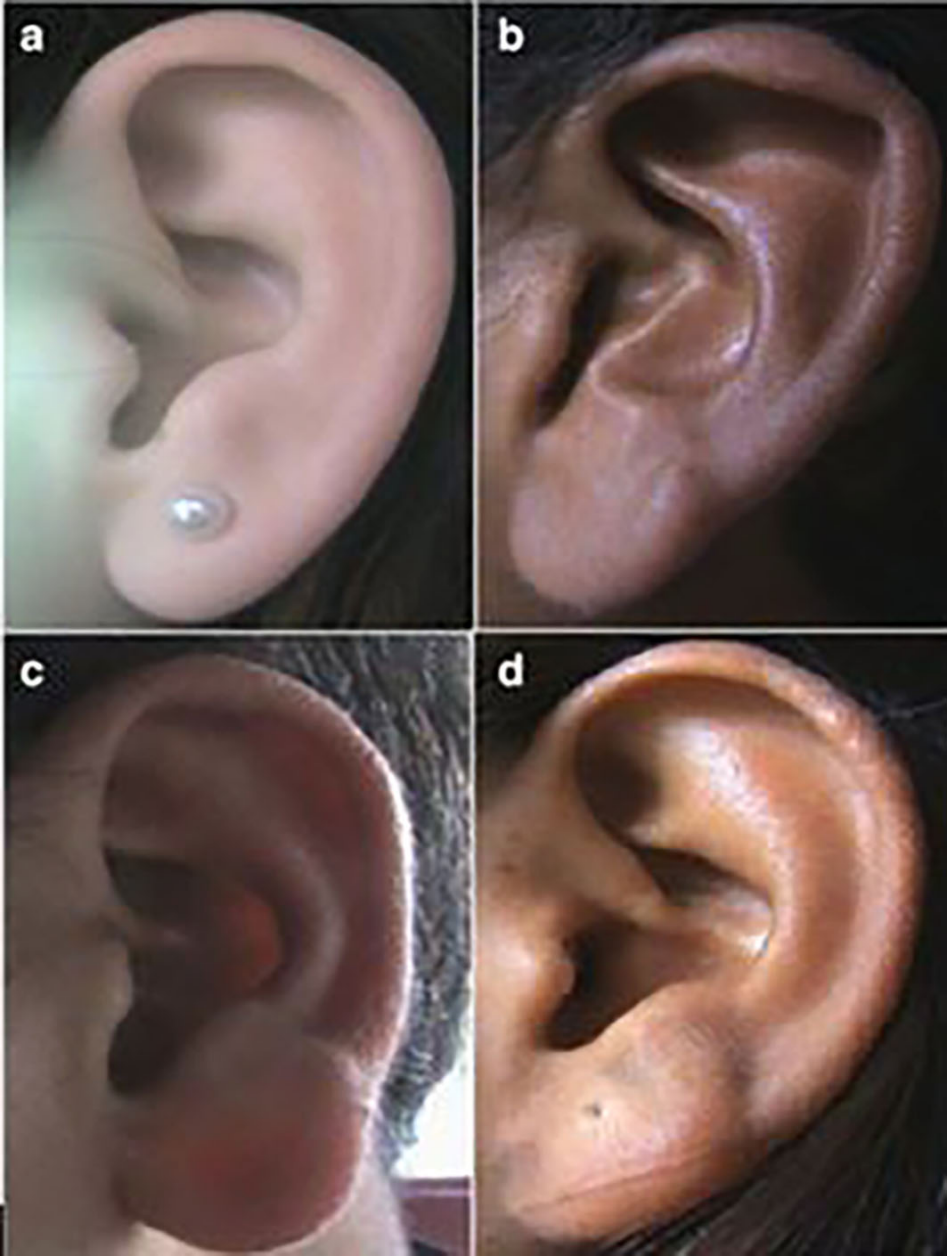
Different types of ear shapes: (a) Oval, (b) Triangular, (c) Rectangular, (d) Round.

**Figure 2.**
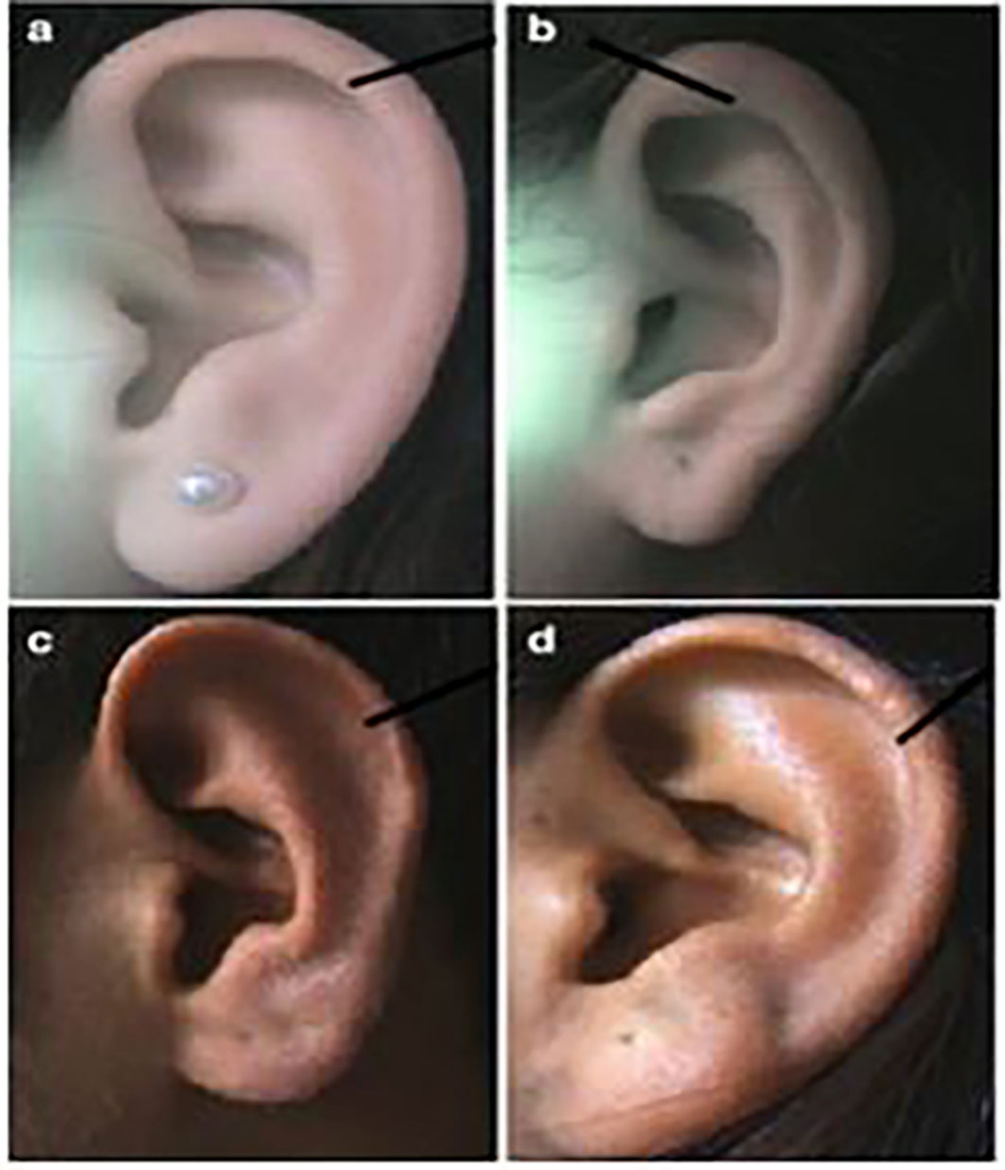
Different forms of helix: (a) normally rolled, (b) wide covering scapha, (c) flat, (d) concave marginal.

**Figure 3.**
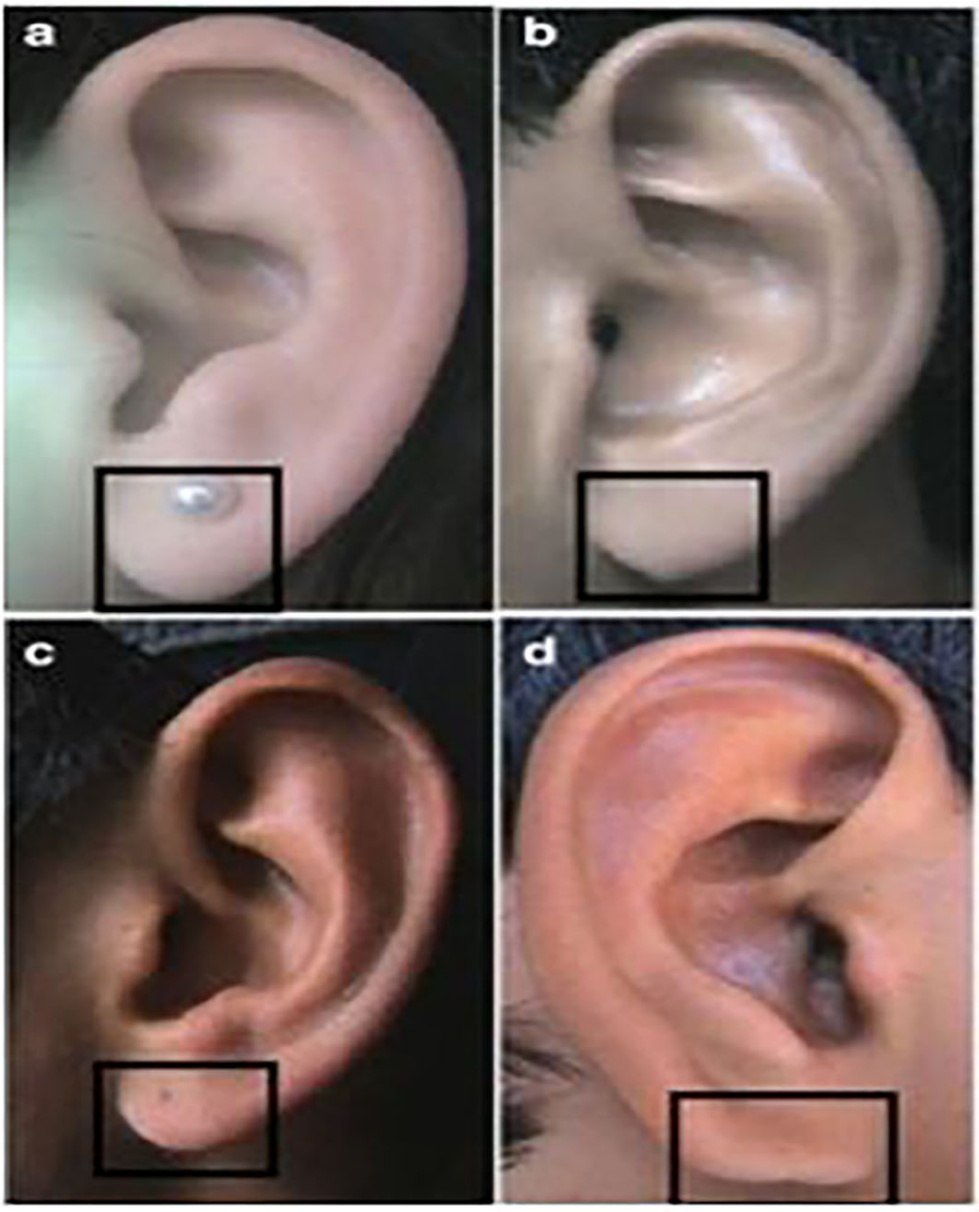
Different types of lobule shapes: (a) Tongue, (b) Triangular, (c) Arched, (d) Square.

**Figure 4.**
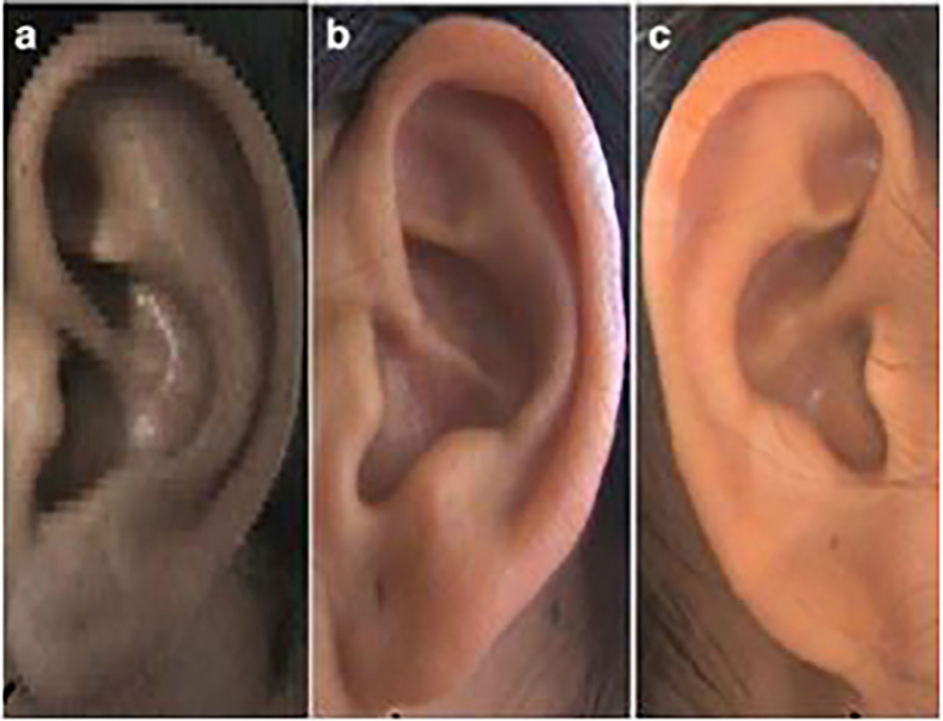
Different types of ear lobe attachment: (a) Free, (b) Partially attached, (c) Attached.

Some research papers have shown variation in ear shapes, ^16^ varied types of helices and tragus, classification of Darwin’s tubercles, and varied types of ear lobes. The types of ear shape are oval, triangular, rectangular, and round. The helix of the ear can take various forms, including flat, gently curved at the edges, broadly covering the scapha, or the more common rolled form. The different lobule shapes are tongue-shaped, triangular, arched, square. The various types of earlobe attachment are classified as free, partially attached, and attached ^17^^–^^20^ ( Figures 1, 2, 3, 4).

Many research papers have found that external ear proportions vary among individuals, and the external ear dimensions are considerably larger in males in addition, ^21^ the ear shows a bilateral asymmetry. Few studies also indicate that the configuration and proportion of the ear can show craniofacial reconstruction in forensic examination. Numerous studies have also reported an association between stature and external ear measurements.

Therefore, the goal of the study is to examine the correlation between the external ear measurements and height, as well as to identify the morphological variation between individuals.

## Methods

The study was conducted on 385 adult male individuals, aged between 18-50 years. The participants were students and professionals of MAHE, Manipal. A total of eight measurements were taken from both ears. The measurements included auricle length, auricle breadth, lobule length, and lobule breadth using a standard caliper, and stature was taken using measuring tape. Participants with deformed ears and female participants (wearing earrings), and also male participants wearing earrings were excluded. Written informed consent was taken from the participants before taking the measurements.

**Figure 5.**
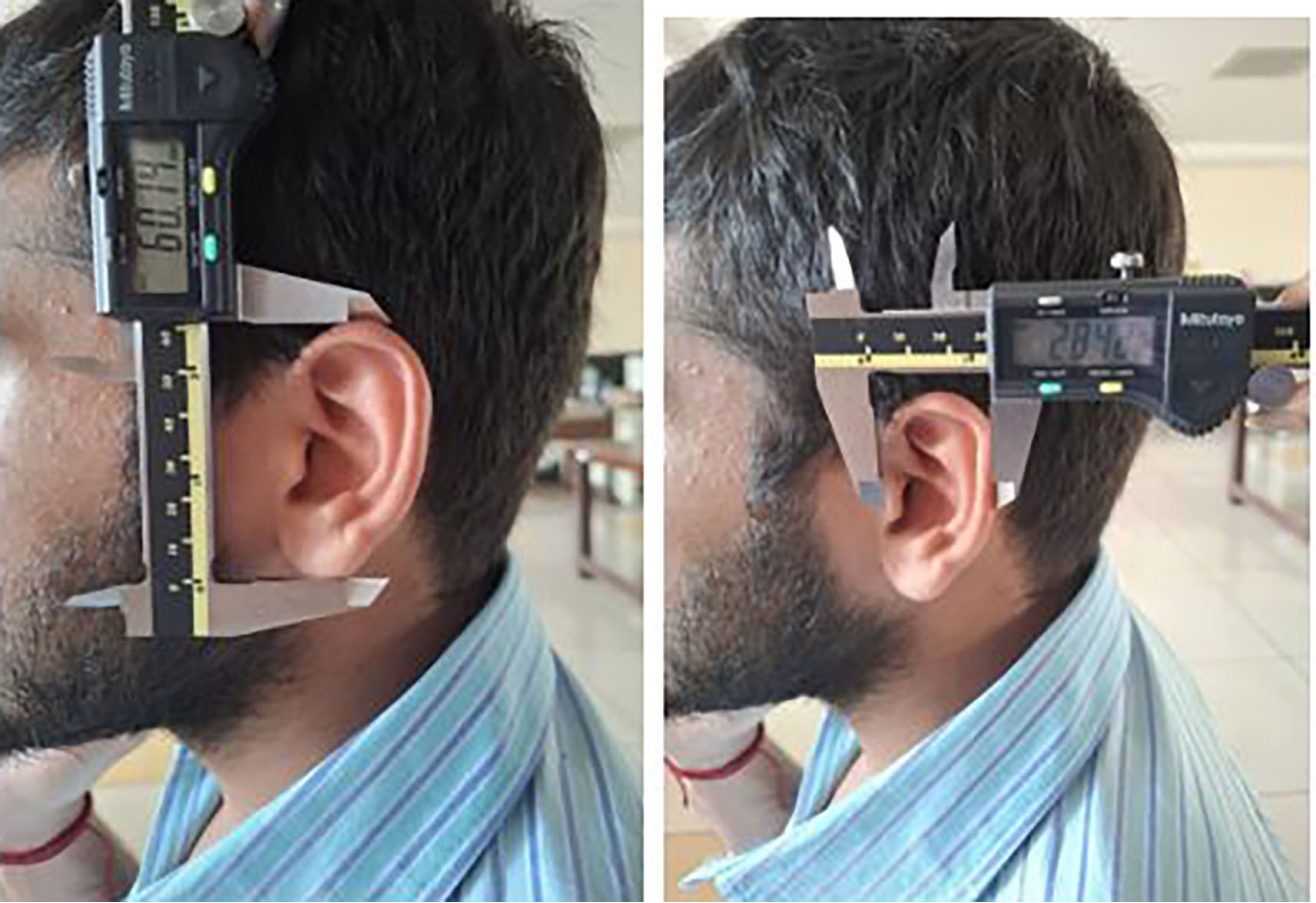
Measuring the ear length and ear width using digital caliper.

**Figure 6.**
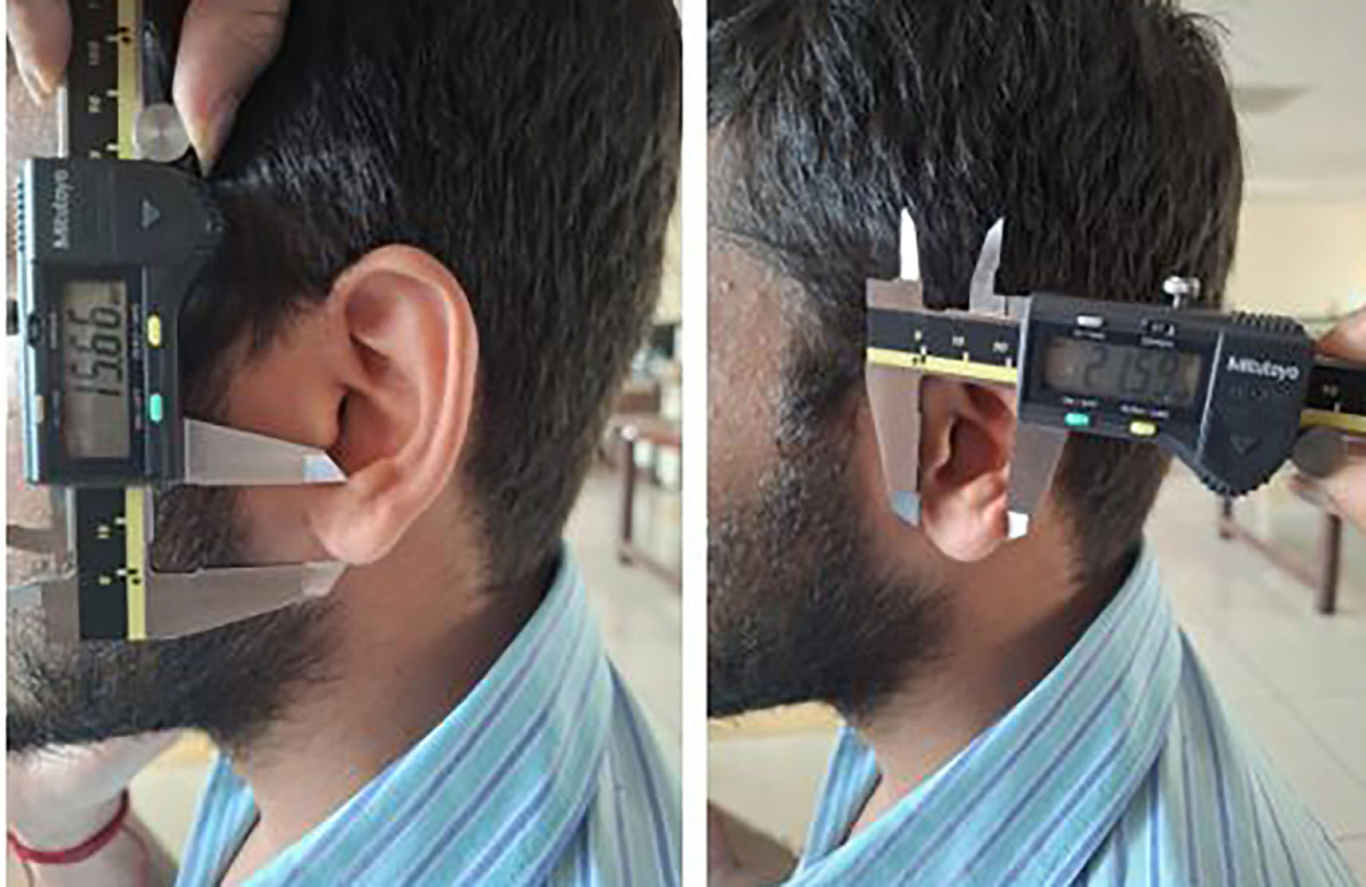
Measuring the lobule length and lobule width using a digital caliper.

Each participant was made to stand barefoot, and the stature was measured using the measuring tape. The participants were made to sit and then the 8 measurements were taken using the digital calliper ( Figures 5, 6).

The data was analysed using Jamovi version 2.4.11. The mean and standard deviation were calculated for all the parameters. Correlation between the stature and the parameters was tested using Spearman’s rank correlation. Linear regression was performed to determine the statistically significant difference between all the parameters and stature.

## Result

From Table 1, the mean and standard deviation of different parameters are age (24.7 ± 6.78), stature (172 ± 6.90), left ear length (6.14 ± 0.462), left ear width (3.22 ± 0.321), left lobule length (2.16 ± 0.350), left lobule width (1.99 ± 0.334), right ear length (7.55 ± 28.6), right ear width (3.24 ± 0.305), right lobule length (2.07 ± 0.336), right lobule width (1.98 ± 0.330). Here, the right ear parameters show a higher mean and standard deviation than the left ear parameters, except for the lobule length and lobule width, where the left side values are higher than the right.

**Table 1.** Descriptive statistics of different parameters (n=385).

PARAMETERS	Mean ± SD
AGE	24.7 ± 6.78
STATURE	172 ± 6.90
LEFT EAR LENGTH	6.14 ± 0.462
LEFT EAR WIDTH	3.22 ± 0.321
LEFT LOBULE LENGTH	2.16 ± 0.350
LEFT LOBULE WIDTH	1.99 ± 0.334
RIGHT EAR LENGTH	7.55 ± 28.6
RIGHT EAR WIDTH	3.24 ± 0.305
RIGHT LOBULE LENGTH	2.07 ± 0.336
RIGHT LOBULE WIDTH	1.98 ± 0.330

SD = Standard Deviation; n = no of individuals.

From Table 2, the result show that in males, the left ear length (R ^2^= 0.0780, p<0.001), left lobule length (R ^2^= 0.00734, p=0.093), left lobule width (R ^2^= 0.0112, p=0.038), right ear length (R ^2^= 0.0660, p=0.072), right ear width (R ^2^= 0.0144, p=0.019), right lobule length (R ^2^= 0.00886, p=0.065), right lobule width (R ^2^= 0.0157, p=0.014) and left ear width (R ^2^=0.00380, p=0.227), among all these parameters left ear length, right ear length shows a statistically significant difference between the stature.

**Table 2.** Statistical analysis of different parameters using linear regression (n=385).

PARAMETERS	R	R ^2^	t	p-value
LEFT EAR LENGTH	0.279	0.0780	5.69	<0.001 *
LEFT EAR WIDTH	0.0617	0.00380	1.21	0.227
LEFT LOBULE LENGTH	0.0857	0.00734	1.68	0.093
LEFT LOBULE WIDTH	0.106	0.0112	2.08	0.038
RIGHT EAR LENGTH	0.257	0.0660	1.84	0.072
RIGHT EAR WIDTH	0.120	0.0144	2.36	0.019
RIGHT LOBULE LENGTH	0.0941	0.00886	1.85	0.065
RIGHT LOBULE WIDTH	0.125	0.0157	2.47	0.014

R - Correlation Coefficient; R ^2^ - Coefficient of Determination; t - t-Statistic.

* p value <0.05- Significant.

From Table 3, the result show that in males, the left auricle length (r= 0.279), left ear width (r= 0.109), left lobule width (r= 0.113), right auricle length (r= 0.279), right ear width (r= 0.130), right lobule width (r= 0.120) shows moderate positive correlation with the stature, while the left lobule length (r= 0.096), right lobule length (r= 0.077) shows weak positive correlation with the stature.

**Table 3.** Statistical analysis of different parameters using Spearman’s rank correlation (data is not normally distributed) (n=385).

PARAMETERS	Spearman’s coefficient (r)	p-value
LEFT EAR LENGTH	0.279	<0.001 *
LEFT EAR WIDTH	0.109	0.033
LEFT LOBULE LENGTH	0.096	0.059
LEFT LOBULE WIDTH	0.113	0.027
RIGHT EAR LENGTH	0.279	<0.001 *
RIGHT EAR WIDTH	0.130	0.010
RIGHT LOBULE LENGTH	0.077	0.130
RIGHT LOBULE WIDTH	0.120	0.019

* p value <0.05- Significant.

From Table 4, the mean age of the 385 individuals was 23 years. The participants were then divided into two groups based on age: those ≤ 23 years and those >23 years. that is lesser than or equal to 23 years of age and greater than or equal to 23 years of age. The two age groups were compared across the eight parameters. The median [IQR] value is taken for all the eight parameters between the two age groups to find which parameter has a significant difference among the individuals based on different age. Among the age ≤23, the left ear length (6.14[5.65-6.31]), left ear width (3.31[3.19-3.39]), left lobule length (1.97[1.81-2.24]), left lobule width (2.05[1.86-2.18]), right ear length (6.06[5.55-6.17]), right ear width (3.43[3.11-3.49]), right lobule length (1.95[1.81-2.20]), right lobule width (2.16[1.80-2.27]) and among the age >23, the left ear length (6.21[5.92-6.40]), left ear width (3.29[3.10-3.50]), left lobule length (2.19[1.90-2.35]), left lobule width (2.15[2.02-2.32]), right ear length (6.14[5.76-6.32]), right ear width (3.29[3.15-3.45]), right lobule length (2.04[1.87-2.43]), right lobule width (2.20[2.01-2.31]).

**Table 4.** Result comparing all parameters between each individual with two age groups using the Mann-Whitney U test (non-normally distributed).

PARAMETERS	AGE	Median[IQR]	p-value
LEFT EAR LENGTH	≤23	6.14[5.65-6.31]	0.371
	>23	6.21[5.92-6.40]	
LEFT EAR WIDTH	≤23	3.31[3.19-3.39]	0.927
	>23	3.29[3.10-3.50]	
LEFT LOBULE LENGTH	≤23	1.97[1.81-2.24]	0.115
	>23	2.19[1.90-2.35]	
LEFT LOBULE WIDTH	≤23	2.05[1.86-2.18]	0.016
	>23	2.15[2.02-2.32]	
RIGHT EAR LENGTH	≤23	6.06[5.55-6.17]	0.329
	>23	6.14[5.76-6.32]	
RIGHT EAR WIDTH	≤23	3.43[3.11-3.49]	0.623
	>23	3.29[3.15-3.45]	
RIGHT LOBULE LENGTH	≤23	1.95[1.81-2.20]	0.120
	>23	2.04[1.87-2.43]	
RIGHT LOBULE WIDTH	≤23	2.16[1.80-2.27]	0.238
	>23	2.20[2.01-2.31]	

IQR- Inter Quartile Range.

Based on the result, the right ear length and left lobule width show a significant difference among the individuals with respect to age. The result shows that as age increases, the right ear length and left lobule width will show a significant difference from person to person.

## Discussion

Human identification is very important in many traumatic events. The main aim of a forensic anthropologist when dealing with bones is to find out the gender, height, years of living, and race. ^22^^–^^24^ The determination of height is necessary in forensic investigation in cases like mutilated human remains or a highly decomposed body. There are several benefits of using somatological dimensions of the outer ear for identification purposes. ^19^ Therefore, the goal of the research was to estimate the stature from the morphological variation of the external auricle and also to evaluate the morphological differences among persons.

In the study by Abdelaleem SA et al., ^25^ a positive correlation was found between all measurements of both ears and stature in both sexes. However, the findings are not in agreement with our study. Archana Kumar et al., ^13^ reported no significant difference for both auricle dimensions in males, which differs from the present findings. In our study, there was a significant difference in left ear length and right ear length with stature.

The key findings of the paper are as follows:

Using the Spearman’s rank correlation, the present study revealed that there was a weak positive correlation between left auricular length (r=0.279, p<0.001) and right ear span (r=0.279, p<0.001) with height.

Estimation of height from the outer auricle dimensions in this paper was performed by using linear regression analysis. The present study revealed that left ear length (R ^2^=0.078), p-value (<0.001) was the best predictable variable to determine the stature, which is in concordance with the findings of Taura MG et al., ^26^ who reported that left ear width (R ^2^=0.086) was the best predictor along with right auricle length (R ^2^=0.082) and left ear length (R ^2^=0.074).

Using the Mann-Whitney U test, the right ear length and left lobule width showed a significant difference between the individuals based on age. The right ear length for age group ≤23 (6.06[5.55-6.17]), >23 (6.14[5.76-6.32]) and left lobule width for the age group ≤23 (2.05[1.86-2.18]), >23 (2.15[2.02-2.32]) showed greater difference in the median [IQR] between the two age group. This indicates that right ear length and left lobule width exhibit morphological variation with age, rather than due to random causes.

To estimate stature from ear morphology, this study highlights the utility of external ear measurements as an additional tool in forensic investigations, in cases of mutilated bodies found in crime scenes, personal identification is important, and when other body parts are not available, then using the ear measurements, we can estimate the stature. ^27^^,^^28^ Although the present findings revealed only a weak positive correlation between stature and ear measurements, the approach may still provide useful information, especially when combined with other methods. These applications might also be beneficial in forensic science, forensic anthropology, and other relevant domains.

### Limitations

In the study, only four parameters were taken from both ears. A manual error may have occurred during the collection of samples. Further studies can include additional parameters, such as facial features, along with the ear parameters. A larger population samples can also be considered for future studies.

## Ethical considerations

The ethical approval IEC No. 397/2024 was granted by “The Institutional Ethics Committee (ICE) of Kasturba Medical College and Kasturba Hospital Manipal” on 13th August 2024. The data were kept completely undisclosed, and no participant identifiers were used. The CTRI approval number is CTRI/2024/10/075400.

## Consent statement

Written informed consent was taken from all the participants. No minors were included in the study.

## Data Availability

Figshare: Stature and external ear morphology. figshare. https://doi.org/10.6084/m9.figshare.28782302.v1. ^29^ Data are available under the terms of the Creative Commons Attribution 4.0 International license (CC-BY 4.0). Extended data: the project contains the following extended data
• Informed consent sheet • Participation information sheet Data can be accessed: Figshare: Stature and external ear morphology_PI and IC forms, https://doi.org/10.6084/m9.figshare.28855265.v1. ^30^ Data are available under the terms of the Creative Commons Zero “No rights reserved” data waiver (CC0 Public domain dedication).
