# Supplementary material for: Deciphering the Morphological Correlation Between Stature and External Ear: An investigative approach in the field of Forensic Science
Source: F1000Res. 2025 Oct 13;14:659. Originally published 2025 Jul 7. [Version 3] doi: 10.12688/f1000research.164138.3 (PMC12596558; doi:10.12688/f1000research.164138.3)
Supplement: Peer review history

# Reviewer report: Reviewer response for version 3

**DOI:** 10.5256/f1000research.189315.r425989

**Published:** 2025-11-20

**Authors:** Erik Schulz

**Affiliations:** 1 University of Nebraska-Lincoln, Lincoln, Nebraska, USA

This paper adds an additional resource for forensic investigators to use in the identification process. To my knowledge there are no current methods that focus on using ear dimensions to develop an estimation of stature. This research can help supplement current methods for identification. The sample size in this study includes just under 400 participants. While this number is not large, it does provide enough evidence to suggest more research and the need for a larger sample size to test the validity of this research. In addition, the studied population is limited to only male participants, as women with earing and males with earrings were excluded. The original study had women participants, but these were later excluded as previous reviewers made mention of females with earrings being excluded, but males with earrings were not. While I agree with this interpretation, I do believe that this publication has been hurt by limiting the populations studied. While conducting a new study may not be feasible, I do believe that it would add more significance to this study if it involved females. I think to remedy this paper, a statement needs to be added to explain why the participants were limited, and that future work will need to be done in order to truly benefit the field of forensics.

Is the work clearly and accurately presented and does it cite the current literature?

Yes

If applicable, is the statistical analysis and its interpretation appropriate?

Partly

Are all the source data underlying the results available to ensure full reproducibility?

Yes

Is the study design appropriate and is the work technically sound?

Partly

Are the conclusions drawn adequately supported by the results?

Yes

Are sufficient details of methods and analysis provided to allow replication by others?

Yes

Reviewer Expertise:

I am a doctoral student in the field of Anthropology with a focus on forensic anthropology and archaeology.

I confirm that I have read this submission and believe that I have an appropriate level of expertise to confirm that it is of an acceptable scientific standard.

# Reviewer report: Reviewer response for version 3

**DOI:** 10.5256/f1000research.189315.r425987

**Published:** 2025-11-08

**Authors:** KUNASILAN SUBRAMANIAM

**Affiliations:** 1 Hospital Kuala Lumpur, Kuala Lumpur, Federal Territory of Kuala Lumpur, Malaysia

Introduction

 The introduction briefly highlights the challenges involved in identifying skeletal remains; however, it does not adequately establish the rationale for using external ear measurements in this context. The authors should clearly articulate how soft tissue parameters, such as external ear dimensions, could meaningfully contribute to biological profiling when skeletal remains are typically devoid of such features.

 It appears more scientifically appropriate to apply external ear measurements in the identification of mutilated but relatively fresh or partially decomposed bodies, where soft tissue features remain intact. This conceptual limitation should be explicitly acknowledged and clarified to avoid misleading the scope of applicability.

 The authors also mention ear size asymmetry but fail to discuss its potential implications for stature estimation. A more thorough explanation is required regarding whether and how bilateral asymmetry could influence measurement precision, regression accuracy, or overall estimation reliability.

 Methodology

 The inclusion of participants aged 18 years and above requires stronger justification. The assumption that stature maturation is complete by 18 years may not hold universally across populations. The authors should provide a supporting reference or rationale indicating that the selected age range represents individuals with stable, fully developed stature.

 The methodology for measuring stature lacks sufficient procedural detail to ensure reproducibility. The authors should specify the exact measuring protocol—whether a stadiometer or measuring tape was used, how the tape was positioned (e.g., affixed to a wall or free-standing), the participant’s posture during measurement, and how intra- and inter-observer errors were minimized. Without such details, replication and methodological reliability cannot be adequately assessed.

 Results

 The reported mean age of 23 years indicates a predominance of younger adults within the sample, with limited representation of older individuals. This skewed age distribution raises concerns regarding the generalizability of the derived regression equations to the wider population. The authors should discuss the extent to which their findings can be extrapolated to older or more diverse demographic groups.

 Discussion

 The discussion section does not sufficiently address the limitations of applying data obtained from living individuals to decomposed or skeletal remains. Given that postmortem changes—such as tissue shrinkage, decomposition, and differential preservation—can significantly affect body length, it is questionable whether stature estimations derived from living subjects are directly transferable to decomposed remains.

 It is recommended that the authors reconsider the stated forensic applicability. Instead of proposing the use of external ear measurements for skeletal or fully decomposed remains, a more plausible and scientifically defensible application would be in the identification of living or recently deceased individuals—particularly in cases where suspects or victims are captured in CCTV footage or other visual recordings. This alternative context would better align with the methodological foundation of the study and enhance its forensic relevance.

Is the work clearly and accurately presented and does it cite the current literature?

Partly

If applicable, is the statistical analysis and its interpretation appropriate?

Partly

Are all the source data underlying the results available to ensure full reproducibility?

Yes

Is the study design appropriate and is the work technically sound?

Yes

Are the conclusions drawn adequately supported by the results?

Partly

Are sufficient details of methods and analysis provided to allow replication by others?

Yes

Reviewer Expertise:

Forensic pathology and forensic anthropology

I confirm that I have read this submission and believe that I have an appropriate level of expertise to confirm that it is of an acceptable scientific standard, however I have significant reservations, as outlined above.

# Reviewer report: Reviewer response for version 3

**DOI:** 10.5256/f1000research.189315.r423285

**Published:** 2025-10-15

**Authors:** Joshua Tetteh

**Affiliations:** 1 Kwame Nkrumah University of Science and Technology, Kumasi, Ghana

Methods: line 4; "Participants with deformed ears and female participants (wearing earrings" please you stated that only male participants wearing ear rings were excluded. I suggest that it should instead read, "Females, and males wearing earrings were excluded.

Is the work clearly and accurately presented and does it cite the current literature?

Partly

If applicable, is the statistical analysis and its interpretation appropriate?

Partly

Are all the source data underlying the results available to ensure full reproducibility?

No

Is the study design appropriate and is the work technically sound?

Yes

Are the conclusions drawn adequately supported by the results?

Partly

Are sufficient details of methods and analysis provided to allow replication by others?

Yes

Reviewer Expertise:

Human Anatomy and Forensic Anthropometry

I confirm that I have read this submission and believe that I have an appropriate level of expertise to confirm that it is of an acceptable scientific standard.

# Reviewer report: Reviewer response for version 2

**DOI:** 10.5256/f1000research.187485.r413038

**Published:** 2025-10-07

**Authors:** Hugo Filipe Violante Cardoso

**Affiliations:** 1 Department of Archaeology & Centre for Forensic Research, Simon Fraser University (Ringgold ID: 1763), Burnaby, British Columbia, Canada

In this paper, the authors aim to explore the relationship between measurements of the external auditory pavilion and living standing stature. The background and overall research design are adequate, but the manuscript requires several substantial revisions before it can be considered for indexing. Below, I outline key areas for improvement.

 1. Justification of the Study

 The manuscript does not provide a strong rationale for the research focus. While the need to estimate stature from different body parts in forensic contexts is acknowledged, two major issues weaken the justification:

- It is unclear why detached ears would be a focus of forensic identification.
- The importance of stature in forensic identification is overstated, particularly since in many jurisdictions there are no reliable ante-mortem records of stature against which to compare estimates.

2. Illustrations and Measurement Descriptions

 The inclusion of illustrations is appreciated, but they require improvement:

- The current figures of ear types do not seem directly relevant to the research question.
- Measurement illustrations should be clearer—either by zooming in on the specific areas of interest or by providing schematic diagrams.
- A detailed description of each measurement is needed. If standardized anthropometric methods were used, the appropriate references should be cited.

3. Methodological Clarifications

 Several methodological issues need attention:

- Avoid repeating the same information in both the text and tables; instead, describe the content of tables without duplicating the data verbatim.
- The age comparison in the results should be justified in the methods section, including an explanation for the choice of 23 years as the cut-off.
- The rationale for using non-parametric tests despite the large sample size needs clarification.
- The interpretation of age effects on ear length is inaccurate: the study does not perform a regression analysis, and therefore cannot assess changes across age. Longitudinal data would be required to answer this question appropriately.

4. Results and Discussion

 The discussion should be expanded to contextualize findings relative to previous studies and to highlight the study’s practical applicability. Specific issues include:

- Regression analysis results are insufficiently reported. If regressions were performed, coefficients, prediction errors, and other relevant statistics should be presented—not just R² values.
- The interpretation of correlations is misleading. Although all correlations are positive, only two are statistically significant, and even these are extremely weak (highest R² = 0.078, explaining only 7.8% of the variation). This suggests that ear measurements are unlikely to be reliable predictors of stature. Statistically significant correlation does not equate to practical predictive value.

5. Study Limitations

 Two important limitations need to be addressed explicitly:

1. **Effect of Age:** There is anecdotal evidence that ear measurements may change with age, but the present data cannot adequately test this. Restricting analyses to a younger subsample might reduce this limitation.
2. **Measurement Error:** The greatest concern is the lack of intra- and inter-observer error assessment. Given the soft tissue nature of the ear, vague landmark definitions, and absence of standardized measurement protocols, error is likely substantial. Without systematic evaluation of measurement error (e.g., repeat measurements by the same and different observers), the reliability of the findings cannot be confirmed.

The manuscript addresses a potentially interesting topic but requires stronger justification, clearer methodology, more rigorous statistical analysis, and explicit consideration of limitations. Incorporating these revisions would substantially improve the clarity, rigor, and overall contribution of the study.

Is the work clearly and accurately presented and does it cite the current literature?

Partly

If applicable, is the statistical analysis and its interpretation appropriate?

Partly

Are all the source data underlying the results available to ensure full reproducibility?

Yes

Is the study design appropriate and is the work technically sound?

Partly

Are the conclusions drawn adequately supported by the results?

Partly

Are sufficient details of methods and analysis provided to allow replication by others?

Partly

Reviewer Expertise:

Forensic and biological anthropology

I confirm that I have read this submission and believe that I have an appropriate level of expertise to confirm that it is of an acceptable scientific standard, however I have significant reservations, as outlined above.

# Reviewer report: Reviewer response for version 2

**DOI:** 10.5256/f1000research.187485.r411585

**Published:** 2025-09-27

**Authors:** Joshua Tetteh

**Affiliations:** 1 Kwame Nkrumah University of Science and Technology, Kumasi, Ghana

1. Introduction: last word should read (individuals), not (individual)

 2. Methods: line 5 should read (were excluded)

 3. Table 1: There must be a legend to show the meaning of SD

 4. Table 2: There must be a legend to show the meaning of R, R ^2 ^and t

 5. Report on table 4: line two; I think it should read those ≤ 23 years and those > 23 years, and line 8 must also read; among the age > 23

 6. Table 4: The age categories in Table 4 should be looked at; it should be ≤ 23 and > 23

 7. Key findings: line 1; the statement should be ... there was a weak positive correlation ... the weak should be present because the correlation values are less than 0.3

 8. Under the Mann-Whitney U test, the age range should be ≤ 23 and > 23

 9. Conclusion: something should be done about the conclusion; the use of ear measurements for stature in the absence of other body parts is too emphatic, but the correlation is weak. The conclusion should therefore be framed to reflect the weak correlation.

Is the work clearly and accurately presented and does it cite the current literature?

Partly

If applicable, is the statistical analysis and its interpretation appropriate?

Partly

Are all the source data underlying the results available to ensure full reproducibility?

No

Is the study design appropriate and is the work technically sound?

Yes

Are the conclusions drawn adequately supported by the results?

Partly

Are sufficient details of methods and analysis provided to allow replication by others?

Yes

Reviewer Expertise:

Human Anatomy and Forensic Anthropometry

I confirm that I have read this submission and believe that I have an appropriate level of expertise to confirm that it is of an acceptable scientific standard, however I have significant reservations, as outlined above.

# Reviewer report: Reviewer response for version 1

**DOI:** 10.5256/f1000research.180598.r403130

**Published:** 2025-08-27

**Authors:** Joshua Tetteh

**Affiliations:** 1 Kwame Nkrumah University of Science and Technology, Kumasi, Ghana

This article aimed to investigate the relationships between the auricle and height, as well as between the two ears. There were significant differences between some left and right ear parameters, and though there were positive correlations between the ear and height, the values are too low to be employed in forensic investigation, as claimed in the conclusion. 

 The work is well presented, but some sentences contain grammatical errors that require attention, especially in the introduction.

 Reference number 30 in the reference list is not found in the text. The arrangement of the in-text references is also not the best (it ends at 22 for the introduction and jumps to 29 in the methods and then comes back to 23 in the discussion)

Is the work clearly and accurately presented and does it cite the current literature?

Partly

If applicable, is the statistical analysis and its interpretation appropriate?

Partly

Are all the source data underlying the results available to ensure full reproducibility?

No

Is the study design appropriate and is the work technically sound?

Yes

Are the conclusions drawn adequately supported by the results?

Partly

Are sufficient details of methods and analysis provided to allow replication by others?

Yes

Reviewer Expertise:

Human Anatomy and Forensic Anthropometry

I confirm that I have read this submission and believe that I have an appropriate level of expertise to confirm that it is of an acceptable scientific standard, however I have significant reservations, as outlined above.

# Response

**Published:** 2025-08-30

**Authors:** Shama das

**Affiliations:** Forensic Medicine,KMC, Manipal Academy of Higher Education, Manipal, Karnataka, India

Dear Reviewer,

 Thank you for your review and inputs. We have incorporated the changes wherever necessary and uploaded the revised version of the manuscript for your kind reference.

- There were significant differences between some left and right ear parameters, and though there were positive correlations between the ear and height, the values are too low to be employed in forensic investigation, as claimed in the conclusion.

Response : We agree with this comment, thank you. However, the conclusion drawn in this research endeavour is based on the significance level. The p values < 0.001 (The present study revealed that left ear length (R ^2^=0.078), p-value (<0.001) was the best predictive variable to determine the stature)  indicate a high level of statistical significance. This finding correlates with findings of a pilot study undertaken by Taura MG et al.in 2016. Hence the research team opines that this finding is of significant value and contributes to the body of knowledge in the domain of forensic sciences.

- The work is well presented, but some sentences contain grammatical errors that require attention, especially in the introduction.

Response: Thank you for this observation. We have incorporated the changes wherever required.

- Reference number 30 in the reference list is not found in the text. The arrangement of the in-text references is also not the best (it ends at 22 for the introduction and jumps to 29 in the methods and then comes back to 23 in the discussion)

Response: Thank you for the observation. We have realigned the references and resubmitted the manuscript for your kind perusal.

- Are all the source data underlying the results available to ensure full reproducibility?

Data Sharing: The underlying data are shared and uploaded to figshare, and cited in the manuscript in the Data Availability section. Request the reviewers to kindly check this section as all underlying data are  made available by the authors. 

 We have addressed all the issues that were mentioned by you. We request you to approve the manuscript for submission.

 Regards,

 Research Team
